# TRGAted: A web tool for survival analysis using protein data in the Cancer Genome Atlas.

**DOI:** 10.12688/f1000research.15789.2

**Published:** 2018-10-01

**Authors:** Nicholas Borcherding, Nicholas L. Bormann, Andrew P. Voigt, Weizhou Zhang

**Affiliations:** 1 Holden Comprehensive Cancer Center, University of Iowa, Iowa City, Iowa, 52245, USA; 2 Department of Pathology, University of Iowa, Iowa City, Iowa, 52245, USA; 3 Cancer Biology Graduate Program, University of Iowa, Iowa City, Iowa, 52245, USA; 4 Medical Scientist Training Program, University of Iowa, Iowa City, Iowa, 52245, USA; 5 Department of Psychiatry, University of Iowa, Iowa City, Iowa, 52245, USA

**Keywords:** Bioinformatics, Cancer Proteomics, Survival Analysis, TCGA

## Abstract

Reverse-phase protein arrays (RPPAs) are a highthroughput approach to protein quantification utilizing antibody-based micro-to-nano scale dot blot. Within the Cancer Genome Atlas (TCGA), RPPAs were used to quantify over 200 proteins in 8,167 tumor and metastatic samples. Protein-level data has particular advantages in assessing putative prognostic or therapeutic targets in tumors. However, many of the available pipelines do not allow for the partitioning of clinical and RPPA information to make meaningful conclusions. We developed a cloud-based application, TRGAted to enable researchers to better examine patient survival based on single or multiple proteins across 31 cancer types in the TCGA. TRGAted contains up-to-date overall survival, disease-specific survival, disease-free interval and progression-free interval information. Furthermore, survival information for primary tumor samples can be stratified based on gender, age, tumor stage, histological type, and subtype, allowing for highly adaptive and intuitive user experience. The code and processed data are open sourced and available on github and contains a tutorial built into the application for assisting users.

## Introduction

Improving prognostic prediction and the identification of potential therapeutic targets is of particular interest to clinicians. Quantification of messenger RNA at a genome-wide level has proven valuable in the discovery of gene expression profiles, which can serve as biomarkers for clinical outcomes in cancer ^1^. However, RNA quantification of tumor or patient cohorts is a proxy for protein level, with many cellular processes above transcription that ultimately regulate protein level. The availability of protein-level quantifications for the TCGA cohort allows for more relevant clinical outcome predictions compared to mRNA levels. Currently, TCGA-based applications provide entry-level analysis in correlational, differential, and survival modalities for the RPPA information. However, survival analysis in these applications rely on median- or mean-based survival data and do not allow for the use of clinical variables ^2– 4^.

**Figure 1.**
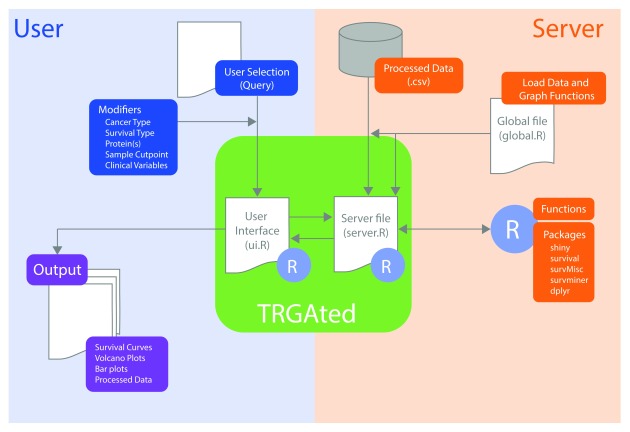
Diagram of the implementation of TRGAted. Each file communicates within the R Shiny framework. On the user side (left, blue), users select pertinent cancer type, protein of interest, and clinical variables into the CSS-enabled user interface. This information is received by the server file enabling the subsequent run in R. On the server side (right, orange), the specific cancer type from the database, R packages, and functions are retrieved and executed. After execution, the server file provides both tabular and graphical output (purple) to the user interface.

With these limitations in mind, we developed a new open-source web application, TRGAted ( Figure 1). Built on the R shiny framework, TRGAted is an intuitive data analysis tool for parsing survival information based on over 200 proteins in 31 cancer types. TRGAted is comprised of processed RPPA information, survival information, and code, allowing users to run instances locally or modify the code with ease.

## Methods

### Protein and survival data

Level 4 TCGA RPPA data for each cancer type was downloaded from the TCPA Portal developed by the MD Anderson Cancer Center ^4^. Across all proteins, individual values were scaled using Z-scores. A summary of information available for each cancer datasets is in Table 1. Additionally, uveal melanoma (UVM) was excluded from the datasets due to a low number of samples with RPPA quantification (n=12). Clinical and survival information for each cancer data set was downloaded from recent work by Liu, *et al.* ^5^. Overall survival, disease-specific survival, disease-free interval, and progression-free interval information was added to primary tumor RPPA quantifications for each cancer type. Unlike other cancer types, metastatic samples were retained for skin cutaneous melanoma (SKCM) RPPA-based dataset due to the highly metastatic nature of the disease. SKCM in the TRGAted application consists of 96 primary tumor samples and 258 metastatic samples. Of the 8,167 samples available in the TCPA, overall survival (OS) data was available for 7,714 patients, disease-specific survival (DSS) data was available for 7,240 patients, disease-free interval (DFI) data was available for 3,887 patients, and progression-free interval (PFI) data was available for 7,315 patients ( Table 1).

**Table 1.** Survival information and protein summary available in TRGAted.

Cancer Type	Samples	OS	DSS	DFI	PFI	Proteins
Adrenocortical carcinoma (ACC)	46	46	46	28	46	221
Bladder Urothelial Carcinoma (BLCA)	344	344	330	153	344	223
Breast invasive carcinoma (BRCA)	901	873	855	750	873	224
Cervical squamous cell carcinoma and endocervical adenocarcinoma (CESC)	171	171	168	112	171	220
Cholangiocarcinoma (CHOL)	30	30	29	21	30	219
Colon adenocarcinoma (COAD)	358	325	311	126	325	223
Diffuse Large B-cell Lymphoma (DLBCL)	33	33	33	19	33	219
Esophageal carcinoma (ESCA)	126	126	124	76	126	220
Glioblastoma multiforme (GBM)	205	136	123	0	136	223
Head and Neck squamous cell carcinoma (HNSC)	346	346	326	85	346	239
Kidney Chromophobe (KICH)	63	63	63	27	63	220
Kidney renal clear cell carcinoma (KIRC)	445	444	434	72	444	233
Kidney renal papillary cell carcinoma (KIRP)	208	207	205	127	207	221
Lower Grade Glioma (LGG)	427	426	420	114	426	220
Liver hepatocellular carcinoma (LIHC)	184	184	177	145	184	220
Lung adenocarcinoma (LUAD)	362	361	327	203	361	239
Lung squamous cell carcinoma (LUSC)	325	325	295	210	325	239
Mesothelioma (MESO)	61	61	45	10	61	220
Ovarian serous cystadenocarcinoma (OV)	411	405	377	199	407	224
Pancreatic adenocarcinoma (PAAD)	105	105	99	40	105	221
Pheochromocytoma and Paraganglioma (PCPG)	81	79	79	71	79	220
Prostate adenocarcinoma (PRAD)	351	351	350	233	351	220
Rectum adenocarcinoma (READ)	130	126	120	31	126	223
Sarcoma (SARC)	221	221	215	125	22	220
Skin Cutaneous Melanoma (SKCM)	354	349	346	0	349	223
Stomach adenocarcinoma (STAD)	392	357	334	207	357	220
Testicular Germ Cell Tumors (TGCT)	118	104	104	79	104	219
Thyroid carcinoma (THCA)	374	372	366	268	372	219
Thymoma (THYM)	90	90	90	9	90	219
Uterine Corpus Endometrial Carcinoma (UCEC)	404	404	403	325	404	223
Uterine Carcinosarcoma (UCS)	48	48	46	22	48	220

OS, overall survival; DSS, disease-specific survival; DFI, disease-free interval; PFI, progression-free interval.

### Implementation

The TRGAted application was written and tested using R v3.5.1. The interactive plots are made using shiny (v1.1.0) and ggplot2 (v3.0.0). Plots can be downloaded as .png, .pdf, or .svg files. Data used to generate the individual plots can be downloaded as .csv files.

*Operation:* Minimum system requirements for running TRGAted locally are modest and include an Intel-compatible CPU and 1 gigabyte of RAM. Running TRGAted from the shiny server requires a modern browser and an internet connection.

**Figure 2.**
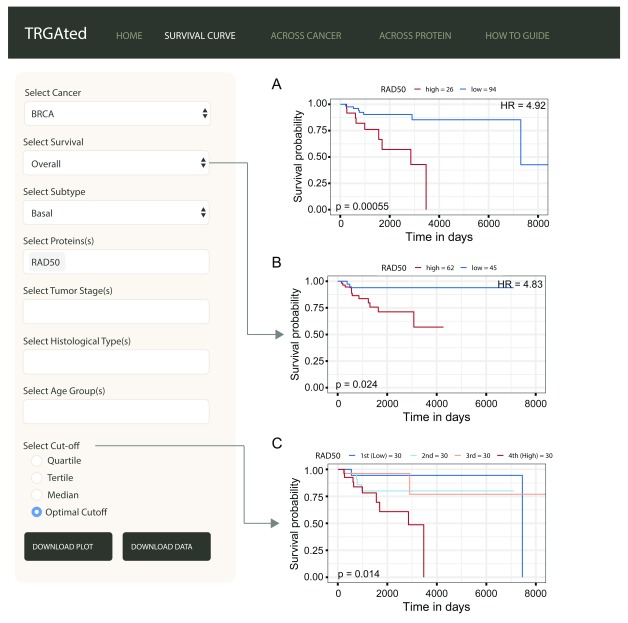
Generating survival curves. The interface shows an example of an overall survival curve for the RAD50 protein in the basal subtype of breast cancer using the optimal cutpoint ( **A**). Disease-specific survival, disease-free interval, and progression-free interval can also be selected ( **B**). The cutpoint can be varied to separate samples based on protein level into quartiles, tertiles, medians or separating into two groups based on the lowest p-value ( **C**).

Kaplan-Meier survival curves can be generated by selecting the cancer type, survival type and protein(s) of interest ( Figure 2). Kaplan-Meier curves are generated using the survival (v2.41-3) and the survminer (v0.4-1) R packages. Multi-protein survival analysis utilizes mean values of protein probes, similar to gene-expression-based survival analysis platforms ^6^. Hazard ratios for two-group comparisons, either median or optimal cut-off, utilize the Cox proportional hazards regression model in the survival R package; with the reported hazard ratio comparing high versus low protein groups. Optimal cut-off feature uses the surv_cutpoint function of the survminer package, calculating the minimal p-value based on the log-rank method. This function uses the maximally selected rank statistic ( maxstat, v0.7-25) R package, which finds the maximal standardized two-sample linear rank statistic ^7^. In order to find clinically or biologically meaningful biomarkers, the minimal proportion cutpoint, or the maximal disparity comparison, was set at 15% versus 85% of samples. Clinical variables dependent on the cancer type selected can be used to filter patients into user-defined groupings. Clinical information available across all types include: subtype, tumor stage, histological type, gender, age, response to primary therapy.

TRGAted also allows for Cox proportional hazard modeling across all proteins in each cancer type or for a single protein across all cancer types. Hazard ratios and p-values are based on the Cox regression model. Values filtered from the volcano plots are proteins with –log10(p-values) less than 0.1 and hazard ratios greater than 20. These filters were implemented to improve visualization and to reduce artifacts of the analysis pipeline, respectively. The volcano plot can be graphed as linear or natural-log transformed, to assist in the visualization of good prognostic indicators. Visualizing the proportional comparison for the volcano plots is also available.

## Use case

In order to demonstrate the functionality of TRGAted, we present a basic survival analysis examining the aggressive, highly-metastatic subtype of breast cancer, known as basal-like breast cancer. We found in this cancer, RAD50, involved in homologous recombination of DNA, as a novel poor prognostic marker.

**Survival curves:** Survival curves can be generated by selecting the cancer type, survival type, and protein or proteins of interest ( Figure 2A). We also selected the subtype information to more closely examine basal-like breast cancer. Other survival types and clinical variables can be selected ( Figure 2B). Samples can be divided into quartiles, tertiles, median or optimally for p-values based on the protein of interest ( Figure 2C). Here we can see that the DNA repair protein, RAD50 is a poor prognostic marker for overall ( Figure 2A) and disease-specific survival ( Figure 2B) in basal-like breast cancer.

**Figure 3.**
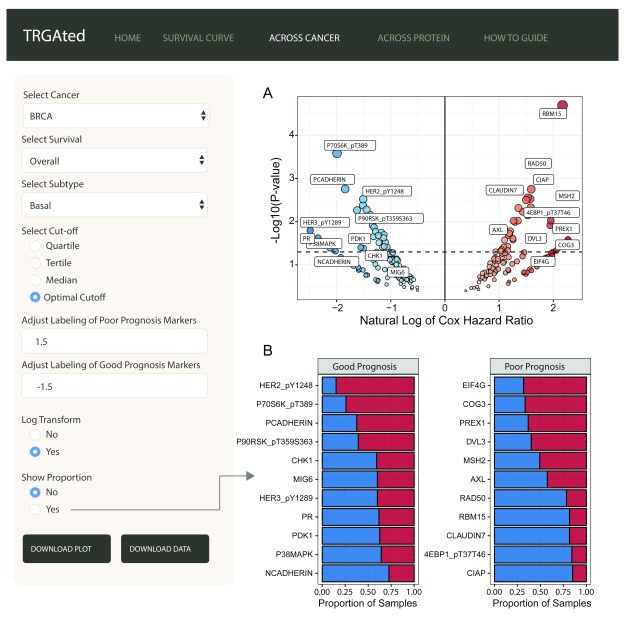
Visualizing all proteins across a single cancer type. The interface shows an example of the visualization of Cox hazard ratio of each protein across the basal subtype of breast cancer ( **A**). Good prognostic markers appear on the left in blue, while poor prognostic markers are on the right in red. The natural log transformation allows the graph to be centered at 0 and makes the visualization of good prognostic markers easier. Labeling for proteins can be adjusted to include more or less protein. Proportional comparisons for protein using the optimal cutpoint function is available as well ( **B**).

**Across cancer:** TRGAted can be used for biomarker discovery by examining the hazard ratios for all proteins available by cancer type or subtype, like basal-like breast cancer ( Figure 3A). The volcano plot displays good prognostic markers on the left in blue and poor prognostic markers on the right in red. Having selected the optimal cutoff feature, a bar chart can also be generated to examine the proportion of samples in the high and low protein groups ( Figure 3B). Protein labeling is adaptive for both the volcano plot and bar chart and will only label significant proteins (p-value ≤ 0.05). Here we see the RAD50 is one of the most significant predictors of poor overall survival in basal-like breast cancer ( Figure 3A and B).

**Figure 4.**
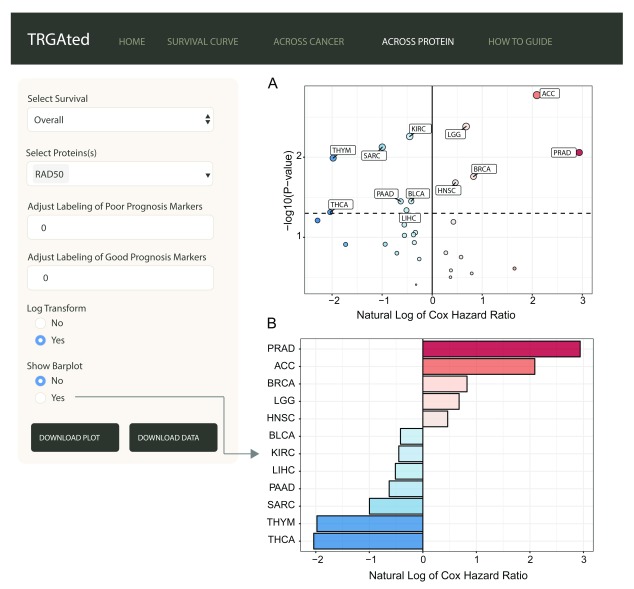
Visualizing all proteins across a single cancer type. The interface shows an example of the visualization of Cox hazard ratio of for RAD50 across all 31 cancer types ( **A**). This feature is similar to the Across Cancer tab with the ability to adjust labels and log-transform the Cox hazard ratios. Additionally, the hazard ratios for significant cancer types can be visualized using a bar chart ( **B**).

**Across protein:** TRGAted can also be used to examine the survival outcomes of a protein of interest across multiple cancers. Here, RAD50 predicts poor survival in only five cancer types, prostate, adrenocortical, breast cancer, low-grade glioma, and head and neck cancers ( Figure 4A). A summary of the hazard ratios can also be visualized by selecting for the barplot function ( Figure 4B).

## Conclusions

TRGAted is an open-source survival analysis application designed to allow for quick and intuitive exploration of TCGA protein-level data. This survival analysis improves on current TCGA pipelines by providing greater diversity of clinical and survival options and relying on protein-level data. In addition to log-rank and Cox regression modeling, TRGAted allows users to download graphical displays and processed data for up to 7,714 samples across 31 cancer types. Built on the R shiny framework, a literate code architecture, the code for TRGAted is annotated and easily modified from our GitHub repository. Under the GNU General Public License v3.0, we encourage interested groups to modify TRGAted for greater usability. Downloading and modifying TRGAted is streamlined by the relatively small size of TRGAted, totally 27.2 megabytes for the application, processed data, and built-in instructional guide.

## Data availability

Release 4.2 of the TCGA replicate-based normalized (level 4) RPPA data is available for 32 cancer types from the TCPA Portal at http://tcpaportal.org/tcpa/download.html. Processed data is available at https://github.com/ncborcherding/TRGAted.

## Software availability

Source code is available from GitHub: https://github.com/ncborcherding/TRGAted/tree/v1.0.0

Archived source code at time of publication: http://doi.org/10.5281/zenodo.1323828 ^8^

License: GNU General Public License v3.0
