# Supplementary material for: TRGAted: A web tool for survival analysis using protein data in the Cancer Genome Atlas
Source: F1000Res. 2018 Oct 1;7:1235. Originally published 2018 Aug 10. [Version 2] doi: 10.12688/f1000research.15789.2 (PMC6173115; doi:10.12688/f1000research.15789.2)
Supplement: Peer review history

# Peer review: Referee response for version 1

**DOI:** 10.5256/f1000research.17235.r38311

**Published:** 2018-09-26

**Authors:** Austin Gillen

**Affiliations:** 1 RNA Bioscience Initiative, University of Colorado School of Medicine, Aurora, CO, USA

In this manuscript, Borcherding, et al. describe an interactive web interface (implemented in R using Shiny) that allows for the visualization of cancer patient survival data from TCGA (The Cancer Genome Atlas) based on protein expression measured by RPPA (Reverse-Phase Protein Array). The software is easy to use, well documented, and flexible enough for most common use cases. The code is available in a public github repository, encouraging further development and expansion of the tool to suit users' needs. There are no major flaws in either the implementation of the tool or the associated manuscript, but two minor issues should be addressed:

 1. As noted by reviewer 1, the manuscript should be carefully proofread for typographical errors and standard english grammar. For example: the common R package ggplot2 is referred to in the text as "ggplots2".

 2. The web interface indicates that the TCPA data included with the package were downloaded on 2017/11/10, but the current TCPA release (4.2) was made available on 2018/07/18. This tool is substantially less useful if it is not updated when new source data is released. A plan for updating the packaged TCPA and survival data should be included in the manuscript (and implemented in the package). Automating this process as a function in the package would be ideal, but detailed instructions for updating the packaged data for local installations would be acceptable as well.

I have read this submission. I believe that I have an appropriate level of expertise to confirm that it is of an acceptable scientific standard.

# Response

**Published:** 2018-09-27

**Authors:** Nicholas Borcherding

**Affiliations:** University of Iowa, USA

We would like to thank the reviewer for the great suggestions. We have recently submitted an updated version of the manuscript with more thorough editing. Additionally, we will work on implementing an automatic pull feature for the data to ensure the most up-to-date protein data available. The specific of this feature will be updated on the github repository and in the application itself when we implement the new pipeline. This was an excellent suggestion.

# Peer review: Referee response for version 1

**DOI:** 10.5256/f1000research.17235.r37844

**Published:** 2018-09-03

**Authors:** Jean Claude Zenklusen

**Affiliations:** 1 National Cancer Institute (NCI), National Institutes of Health (NIH), Bethesda, MD, USA; 2 Clinical Commissioning Groups (CCG) , London, UK; 3 National Institutes of Health (NIH) Clinical Center, Bethesda, MA, USA

This report by Borcherding et al. deals with the creation of a tool to visualize the impact of proteins represented in the Reverse Phase Protein Array (RPPA) on the survival of patients used in The Cancer Genome Atlas (TCGA). The tools are straight forward, uses a common standard (it is an R module) and thus has the potential of being highly utilized by the cancer research community. There are no major flaws with the module, the code is deposited in github, allowing easy access to users.

 Two minor issues need to be corrected:

1. The manuscript will benefit from editing by a native English speaker. Phrasing and grammar are uncommon at times.
2. Reference 5 is referred as “updated TCGA clinical data”. This is incorrect. The paper referred to is an interpretation of the clinical data in the context of the Pan Can Atlas effort by the TCGA, but it is NOT the official clinical data. It is a derived product of it.

I have read this submission. I believe that I have an appropriate level of expertise to confirm that it is of an acceptable scientific standard.

# Response

**Published:** 2018-09-27

**Authors:** Nicholas Borcherding

**Affiliations:** University of Iowa, USA

Thank you for your very kind review and suggestions. In the most recent submission, we have addressed your concerns in editing the manuscript and adding additional details on the source of clinical information.
